# Subsurface ocean flywheel of coupled climate variability in the Barents Sea hotspot of global warming

**DOI:** 10.1038/s41598-019-49965-6

**Published:** 2019-09-23

**Authors:** Pawel Schlichtholz

**Affiliations:** Institute of Oceanology, Polish Academy of Sciences, Powstancow Warszawy 55, 81-712 Sopot, Poland

**Keywords:** Atmospheric dynamics, Cryospheric science, Physical oceanography

## Abstract

Accelerated shrinkage of the Arctic sea ice cover is the main reason for the recent Arctic amplification of global warming. There is growing evidence that the ocean is involved in this phenomenon, but to what extent remains unknown. Here, a unique dataset of hydrographic profiles is used to infer the regional pattern of recent subsurface ocean warming and construct a skillful predictor for surface climate variability in the Barents Sea region - a hotspot of the recent climate change. It is shown that, in the era of satellite observations (1981–2018), summertime temperature anomalies of Atlantic water heading for the Arctic Ocean explain more than 80% of the variance of the leading mode of variability in the following winter sea ice concentration over the entire Northern Hemisphere, with main centers of action just in the Barents Sea region. Results from empirical forecast experiments demonstrate that predictability of the wintertime sea ice cover in the Barents Sea from subsurface ocean heat anomalies might have increased since the Arctic climate shift of the mid-2000s. In contrast, the corresponding predictability of the sea ice cover in the nearby Greenland Sea has been lost.

## Introduction

The Arctic climate system is particularly vulnerable to global warming instigated by anthropogenic increases of carbon dioxide and other greenhouse gases^1–3^. Dramatic changes have been recently observed in all components of the Arctic climate system, including the polar atmosphere^4–6^, cryosphere^7–9^ and the Arctic Ocean interior^10–13^. These changes may have potentially far-reaching consequences not only for Arctic ecology and human activities in the high North^14^ but also for extreme weather events and climate anomalies at lower latitudes^15–22^. Over recent decades, surface atmospheric warming in the Arctic has occurred at a rate that is at least two times higher than globally^5,16,23^. This phenomenon, known as Arctic amplification, is most pronounced during the ice-growth season (autumn and winter). Arctic amplification has risen above the level of climate noise in the late 1990s^16^. Its apparent emergence at that particular time might have been related to a temporary slowdown in the surface temperature warming trend over the rest of the globe^16,24^. This slowdown, if at all significant^25^, did not represent a reduced pace in warming of the climate system. It rather reflected a modulation of global surface warming by external drivers other than anthropogenic forcing, warming of increasingly deeper layers of the ocean, and redistribution of energy within the oceans by natural modes of climate variability that changed their phase in the 1990s^24,26–29^. In fact, all oceans have experienced significant basin-averaged warming since 1998^28^. In any case, surface global warming has accelerated in recent years^30,31^ due to a further enhanced air temperature rise in the Arctic^19,30,32^.

**Figure 1:**
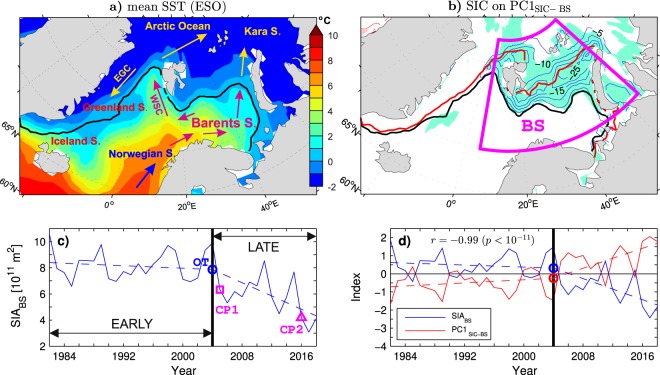
Variability of the winter (DJF) mean sea ice cover in the Barents Sea region during the ESO period. (**a**) Climatological sea ice edge (15% SIC contour, black line) on the background of the climatological mean SST (colours) obtained by averaging data over all ESO winters. The arrows with acronyms depict the West Spitsbergen Current (WSC) and the East Greenland Current (EGC). (**b**) (thin contours and colour shading) Undetrended SIC anomalies regressed onto the principal component time series (PC1_SIC−BS_) of the leading EOF mode of the SIC variability in the Barents Sea region (BS box) and (thick lines) the mean 15% SIC contour in winters 2003/04 (black line) and 2017/18 (red line). The thin blue (resp. red) contours represent negative (resp. positive) anomalies. The contour interval (CI) is 5% per unit PC1_SIC−BS_. The zero contour is omitted. Aquamarine (resp. pink) shading marks negative (resp. positive) anomalies statistically significant at the 95% confidence level. (**c**) (solid blue curve) Time series of the sea ice area in the Barents Sea region (SIA_BS_ index) and (dashed blue line) its continuous piecewise linear trend with the breakpoint in winter 2003/04. The blue circle, magenta square and magenta triangle mark the onset time (OT) of the sea ice decline, and the first (CP1) and second (CP2) regime change points, respectively (see Methods). (**d**) (solid curves) Standardised time series of SIA_BS_ (blue curve) and PC1_SIC−BS_ (red curve), and their OT points (circles) and continuous piecewise linear trends with the breakpoint in winter 2003/04 (dashed lines). In (**a**,**b**) the maps were generated by MathWorks MATLAB R2014a with M_Map (http://www.eoas.ubc.ca/rich/map.html). In (**c**,**d**) each year on the horizontal axis includes January of the DJF season.

While several atmospheric processes may contribute to Arctic amplification^33–37^, this phenomenon is profoundly related to the diminishing Arctic sea ice cover via the ice-albedo and other effects^23,38^. The sea ice loss is most spectacular in summer, at the end of the melt season, but is also significant in other seasons^8,9,39^. In the Barents Sea (see Fig. 1a for its location), the reduced sea ice concentration (SIC) between winters 2002/03 and 2014/15 resulted in a spectacular rise of local surface air temperature (SAT) by up to 20 °C^6^. There are indications that remote changes in sea surface temperature (SST) may control Arctic warming via their influence on atmospheric circulation^40,41^. There is also growing evidence that changing inflows of warm and salty Atlantic water (AW) to the Barents Sea and the Arctic Ocean contribute to the shrinkage of the Arctic sea ice cover^11,12,42,43^. The wintertime sea ice decline is consistent with recent warming signals detected in the Barents Sea^13,44,45^ as well as in the pathway of AW to the Arctic Ocean through Fram Strait^46,47^, and then eastward along the Arctic Ocean margin^12,48^.

The ocean “memory” due to the large heat capacity of seawater combined with long ocean advective timescales make the Arctic sea ice changes, especially those in the Barents Sea, to some extent predictable form upstream oceanic conditions^49–51^. Remarkably high predictability of the total wintertime sea ice area (SIA) in the Barents and Nordic (Greenland-Iceland-Norwegian) Seas region from summertime anomalies of Atlantic water temperature (AWT) was reported from an analysis of observations in the period 1982–2006^49^. In that period, the ocean acted as a coordinator of the regional SIC variability. Indeed, the oceanic impact on the sea ice cover in the Barents Sea and the Greenland Sea considered separately was not as strong as its impact on the coherent SIC variability in these seas^49^. The main objective of the present study is to verify the hypothesis that the high predictability of the sea ice cover in the Barents/Nordic Seas region from AWT anomalies survived through the most recent changes in the Arctic climate system. This survival is not a priori granted since a sharp sea ice decline observed in the northern Barents Sea in the mid-2000s^52,53^ could be linked to reduced wintertime sea ice import from the nearby Kara Sea^13,54^, which could limit the ability of AWT anomalies to control the sea ice extent in the Barents Sea.

Here, the variability of the Arctic climate system is investigated using statistical analyses applied to SIC, SST and atmospheric fields, as well as subsurface ocean data covering the era of satellite observations (ESO period) from 1981 to 2018. In addition to indices based on area-averaged quantities, indices of climate variability are derived using the empirical orthogonal function (EOF) technique (see Methods). A unique subsurface ocean dataset is used to analyse the recent ocean warming in the Barents/Nordic Seas region and construct a reliable AWT time series employed in empirical forecast experiments (see Methods). This dataset consists of ocean temperature profiles from the newly compiled Unified Database for Arctic and Subarctic Hydrography (UDASH)^55^ supplemented with *in situ* temperature observations from other sources (see Methods).

## Variability of the Wintertime Sea Ice Cover in the Barents Sea Region

Variability of the wintertime sea ice cover in the Barents Sea region is characterised by the December-February (DJF) mean SIA_BS_ index defined as the ocean area covered by sea ice within the BS box in Fig. 1b. The box encompasses the Barents Sea itself (the area between Norway, Spitsbergen, Franz Joseph Land and Novaya Zemlya), the West Spitsbergen Current (see the arrows in Fig. 1a for a schematic representation of the regional ocean circulation plotted on the background of the wintertime SST climatology), and the area along the Arctic Ocean margin north of Svalbard and the Barents Sea shelf. During the EARLY period (winters 1981/82–2003/04), the time series of SIA_BS_ exhibits considerable interannual variability but practically no trend (Fig. 1c, blue curve). In contrast, the interannual variations of SIA_BS_ in the LATE period (winters 2003/04–2017/18) appear as departures from a marked long-term decline (the linear trend significant at *p* = 0.02). Through the LATE period, SIA_BS_ declined by as much as 58% (from 9.8 × 10^11^ to 4.1 × 10^11^ m^2^). The decline occurred in three winter-to-winter pulses, first from 2003/04 to 2004/05, then from 2010/11 to 2011/12 and finally from 2014/15 to 2015/16, separated by events of recoveries to relatively heavy ice conditions. The decline culminated with unprecedentedly low values of SIA_BS_ in the last three (LAST3) winters (2015/16–2017/18), with a record of −2.5 standard deviations in 2016/17.

In order to objectively identify the onset time (OT) of recent changes in the Barents Sea ice cover and other Arctic climate variables, an algorithm for detection of nonlinear transitions is employed. The algorithm is based on data departures from a simplified form of the given time series^54^ (see Methods). In SIA_BS_, the OT point of the sea ice decline (marked by a blue circle in Fig. 1c) is found in winter 2003/04. The change in SIA_BS_ from this winter to the following one (2004/05) is qualified as a regime shift using a regime change detection method. The method is based on a comparison of means in sliding segments and subsequent data in the given time series^56^ (see Methods). According to this method, also the abrupt sea ice decline from 2014/15 to 2015/16 can be qualified as a regime shift. In Fig. 1c, the points of the first (CP1) and second (CP2) detected abrupt changes in the mean of SIA_BS_ are marked by a square and triangle, respectively. Two regime shifts during the ESO period, one at the transition between the EARLY and LATE periods and one leading to abnormal conditions in the LAST3 winters are also identified in other indices of Arctic climate variability. Results of the search for the CP and OT points in selected indices are included in Table 1, which also reports other statistics, such as the statistical significance of linear trends in the LATE period (column *p*_LATE_). Whether the transition near the end of the LATE period is a genuine regime shift or an interannual bump in the time series cannot be determined at present.

**Table 1:** Statistics of indices of the winter (DJF) mean Arctic climate variability and their subsurface ocean predictor during the ESO period (1981–2018).

Index	Figure	CP1	CP2	OT	OT_11_	*p*_LATE_	*r*_AWT_	*p*_Δ*r*_
SIA_BS_	1c	2004/05	2015/16	2003/04	2000/01	0.017	−0.88	5 × 10^−3^
PC1_SIC−BS_	1d	2004/05	2015/16	2003/04	2000/01	0.014	0.87	3 × 10^−3^
SST_sBS_	2c	2004/05	2015/16	2003/04	2000/01	0.012	0.88	2 × 10^−4^
PC1_SST−BS_	2d	2004/05	2015/16	2003/04	2000/01	0.005	0.88	2 × 10^−3^
PC1_SIC−A40_	3c	2004/05	2015/16	1997/98	2000/01	0.001	0.90	9 × 10^−2^
PC1_SAT−A70_	3d	2004/05	2015/16	2003/04	1995/96	0.009	0.79	3 × 10^−1^
SAT_nBS_	3d	2004/05	2015/16	2003/04	1994/95	0.038	0.79	1 × 10^−2^
SIA_BNS_	4f	2004/05	2015/16	1997/98	2000/01	0.040	−0.92	1 × 10^−1^
AWT_SSS_	4f	2004	2015	1997	2000	0.025	—	—

**Figure 2:**
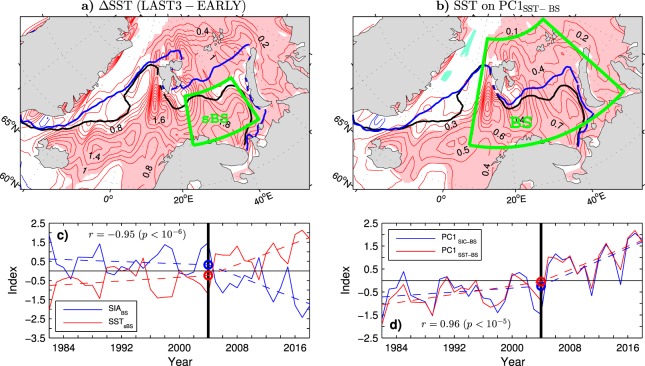
Relationship between the winter mean sea surface temperature and sea ice concentration in the Barents/Nordic Seas region during the ESO period. (**a**) (thin contours and colour shading) Difference in the mean SST between the last three (LAST3) winters (2015/16–2017/18) and the winters of the EARLY period (1981/82–2003/04), and (thick lines) the 15% contour of the mean wintertime SIC in the LAST3 winters (blue line) and the EARLY period (black line). (**b**) (thin contours and colour shading) Undetrended SST anomalies regressed onto the principal component time series (PC1_SST−BS_) of the leading EOF mode of the SST variability in the Barents Sea region (BS box) in the ESO period and (thick lines) the mean 15% SIC contour in the EARLY period (black line) and the LATE period (winters 2003/04–2017/18, blue line). (**c**) (solid curves) Standardised time series of (red curve) the average SST over the southern Barents Sea region (sBS box in **a**) and (blue curve) sea ice area in the Barents Sea region (SIA_BS_ index), and their OT points (circles) and continuous piecewise linear trends with the breakpoint in winter 2003/04 (dashed lines). (**d**) (solid curves) Standardised time series of (red curve) PC1_SST−BS_ and (blue curve) PC1_SIC−BS_, and their OT points (circles) and continuous piecewise linear trends with the breakpoint in winter 2003/04 (dashed lines). In (**a**,**b**) the thin contour and shading colours are explained in the caption to Fig. 1. The CI is 0.2 °C and 0.1 °C per unit PC1_SST−BS_, respectively. The maps were generated by MathWorks MATLAB R2014a with M_Map (http://www.eoas.ubc.ca/rich/map.html). In (**c**,**d**) each year on the horizontal axis includes January of the DJF season.

**Figure 3:**
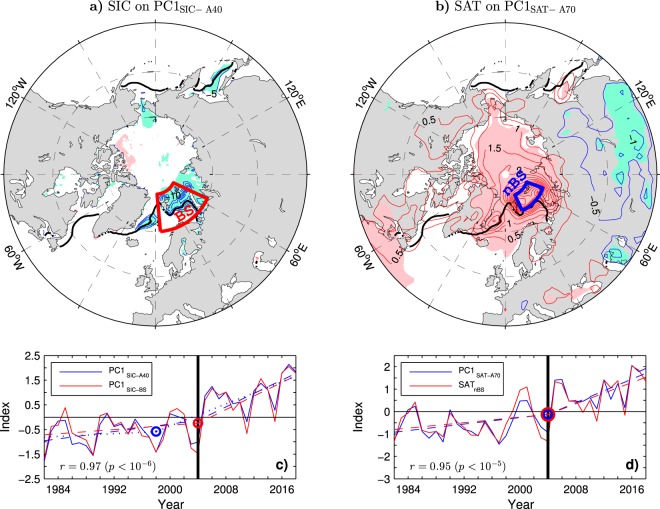
Leading modes of the variability in the winter mean Arctic sea ice concentration and surface air temperature during the ESO period. (**a**) Undetrended SIC anomalies regressed onto the principal component time series (PC1_SIC−A40_) of the leading EOF mode of the SIC variability north of 40°N. (**b**) Undetrended SAT anomalies regressed onto the principal component time series (PC1_SAT−A70_) of the leading EOF mode of the SAT variability north of 70°N. (**c**) (solid curves) Standardised time series of (blue curve) PC1_SIC−A40_ and (red curve) PC1 of the SIC variability in the Barents Sea region (BS box in **a**), their OT points (circles)and continuous piecewise linear trends with the breakpoint in winter 2003/04 (dashed lines), and (for PC1_SIC−A40_ only) the continuous piecewise linear trend with the breakpoint in winter 1997/98 (dotted line). (**d**) (solid curves) Standardised time series of (blue) PC1_SAT−A70_ and (red) the average SAT over the northern Barents Sea region (nBS box in **b**), and their OT points (circles) and continuous piecewise linear trends with the breakpoint in winter 2003/04 (dashed lines). In (**a**,**b**) the thin contour and shading colours are explained in the caption to Fig. 1. The CI is 5% per unit PC1_SIC−A40_ and 0.5 °C per unit PC1_SAT−A70_, respectively. The thick black lines indicate the climatological mean wintertime ice edge (15% SIC contour). The maps were generated by MathWorks MATLAB R2014a with M_Map (http://www.eoas.ubc.ca/rich/map.html). In (**c**,**d**) each year on the horizontal axis includes January of the DJF season.

**Figure 4:**
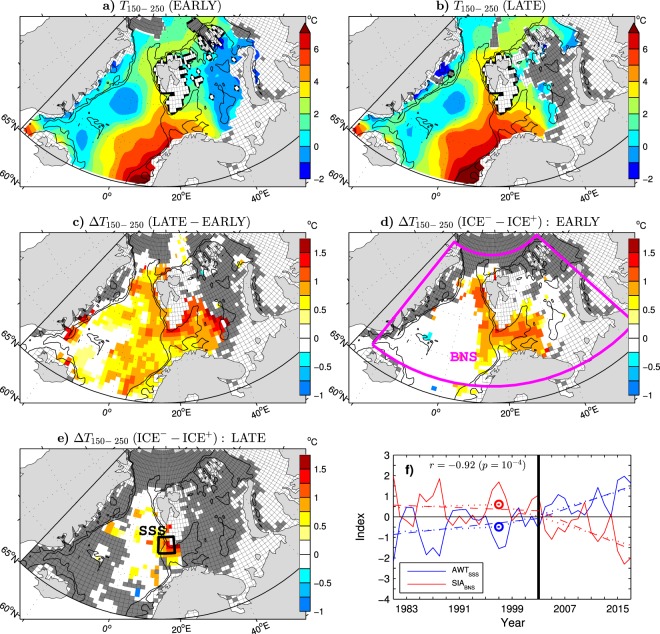
Relationship between the summer (JJA) mean subsurface ocean temperature and the following winter sea ice area in the Barents/Nordic Seas region during the ESO period. (**a**) (colours) Climatological mean temperature averaged over the 150–250 m layer (*T*_150−250_) in the EARLY period (summers 1981–2003). (**b**) As in (**a**) but for the LATE period (summers 2004–2017). (**c**) (colours) Difference in the mean of *T*_150−250_ (in °C) between the LATE and EARLY periods. (**d**) (colours) Difference in the composite mean of *T*_150−250_ between six summers with the smallest and six summers with the largest sea ice coverage in the Barents/Nordic Seas region marked as BNS box (SIA_BNS_ index) in the following winter during the EARLY period. (**e**) As in (**d**) but for the summers preceding three winters with the smallest and four winters with the largest SIA_BNS_ during the LATE period. (**f**) (solid curves) Standardised time series of (blue curve) the summer mean Atlantic water temperature averaged over the southern Svalbard slope area (SSS box in **e**) and (red curve) the following winter SIA_BNS_ index, their OT points (circles), their continuous piecewise linear trends with the breakpoint in summer 1997 and winter 1997/98 (dotted lines), and their continuous piecewise linear trends with the breakpoint in summer 2003 and winter 2003/04 (dashed lines), respectively. Each year on the horizontal axis refers to the summer season. In (**a**–**e**) grid lines are masked in the areas of valid data. Grid cells shallower than 150 m are white shaded, while areas of missing ocean data are dark shaded. In (**c**–**e**) differences smaller than 0.2 °C or nonsignificant at the 95% confidence level are white shaded. The maps were generated by MathWorks MATLAB R2014a with M_Map (http://www.eoas.ubc.ca/rich/map.html).

The PC1s (PC1_SIC−BS_, PC1_SST−BS_, PC1_SIC−A40_ and PC1_SAT−A70_) are for the sea ice concentration (SIC) and the sea surface temperature (SST) in the Barents Sea region (BS box in Fig. 1b), hemispheric SIC north of 40°N (A40 area), and surface air temperature (SAT) in the high Arctic north of 70°N (A70 area). The other wintertime indices represent the concurrent area-averaged SST in the southern Barents Sea (sBS box in Fig. 2a), area-averaged SAT in the northern Barents Sea area (nBS box in Fig. 3b), sea ice area (SIA) in the Barents Sea region, and SIA in the Barents/Nordic Seas region (BNS box in Fig. 4d). The subsurface ocean predictor is the summer (JJA) mean Atlantic water temperature (AWT) in the southern Svalbard slope area (SSS box in Fig. 4e). Columns CP1 and CP2 list years of the first and second regime change in the mean of the time series, respectively. Columns OT and OT_11_ give onset times (years) of the sea ice decline or oceanic/atmospheric warming based on the raw time series and their filtered version (11-yr running mean), respectively. Column *p*_LATE_ reports *p*-values from a one-sided *t*-test for a non-zero linear trend in the LATE period (winters 2003/04–2017/18 or summers 2003–2017 in the case of AWT_SSS_). Column *r*_AWT_ gives correlation coefficients between the given wintertime index and the preceding summer AWT_SSS_ during the entire ESO period. All correlations are significant at the 99.8% or higher confidence level. Column *p*_Δ*r*_ lists empirical *p*-values from a Monte Carlo test for the difference between respective correlations in the EARLY period (winters 1981/82–2003/04) and the LATE period. Details of the CP1, CP2, OT and OT_11_ detection are outlined in Methods.

In the LATE period, a sharp sea ice decline took place north and northeast of Svalbard. In this area, a quasi-steady reopening and eastward progression of a wintertime polynya (open water zone), known as the Whalers Bay, was observed^48^. A remarkable sea ice retreat was also observed in the northern Barents Sea^13,52^. The recent sea ice decline in these areas is illustrated by thick black and red lines in Fig. 1b depicting the mean location of the ice edge (15% SIC contour) in the Barents/Nordic Seas region during winters 2003/04 and 2017/18, respectively. The same figure also shows SIC anomalies associated with the principal component time series (PC1_SIC−BS_) of the leading EOF mode of the winter mean SIC variability in the Barents Sea region during the ESO period (see thin blue contours for negative SIC anomalies and aquamarine shading for areas where these anomalies are significant at the 95% confidence level). This mode explains 62% of the regional SIC variance. Its temporal variability is practically indistinguishable from the variability of the area-averaged sea ice cover in the Barents Sea (*r* = −0.99; see Fig. 1d for comparison of the standardised time series of PC1_SIC−BS_ and SIA_BS_). The correlation between PC1_SIC−BS_ and SIA_BS_ is negative because the upward trend in PC1_SIC−BS_ corresponds to the sea ice decline (negative SIC anomalies in Fig. 1b).

## Relation to Concurrent Anomalies of the Sea Surface Temperature

The recent sea ice retreat in the Barents Sea region was accompanied by ocean warming, as shown by the pattern of the wintertime SST difference between the means in the LAST3 years and the EARLY period (see Fig. 2a; thin red contours for positive SST differences and pink shading for areas where these differences are significant at the 95% confidence level). In the area of the sea ice retreat (see thick blue and black lines in Fig. 2a for the mean location of the ice edge during the LAST3 and EARLY period winters, respectively), local SST warming exceeded 1 °C from the northeastern tip of Spitsbergen to the passage between Novaya Zemlya and Franz Joseph Land. In the open water, the warming reached 2 °C in the southern Barents Sea and southern part of the West Spitsbergen Current. Significant open water warming of up to 1.5 °C is also observed upstream, in the Norwegian Sea. An index of open water SST variability (SST_sBS_ index) is defined as SSTs averaged over the southern Barents Sea (sBS box in Fig. 2a). Like the sea ice cover in the Barents Sea, the wintertime SST_sBS_ index exhibits a nonsignificant trend (*p* > 0.1) during the EARLY period followed by a significant trend (*p* = 0.01) during the LATE period (see Fig. 2c for comparison of the standardised time series of SST_sBS_ and SIA_BS_). In the LATE period, this index is characterised by winter-to-winter warming pulses that coincide with the pulses of the Barents Sea ice decline. Over the entire ESO period, SST_sBS_ correlates with SIA_BS_ at a high level (*r* = −0.94), suggesting that the wintertime sea ice extent in the Barents Sea region is controlled by SST anomalies.

The pattern of the wintertime SST difference between the LAST3 years and the EARLY period (Fig. 2a) is strikingly similar to the pattern of SST anomalies associated with the principal component time series (PC1_SST−BS_) of the leading EOF mode of the winter mean SST variability in the Barents Sea region (BS box in Fig. 2b) during the ESO period (Fig. 2b, thin contours and colour shading). This mode explains 60% of the regional SST variance and is strongly coupled to the concurrent variability in the Barents Sea ice cover. PC1_SST−BS_ correlates highly with SIA_BS_ (*r* = −0.95, *p* < 10^−5^) and PC1_SIC−BS_ (*r* = 0.96; see Fig. 2d for comparison of the time series). These relations remain equally strong (|*r*| ≈ 0.95) after removal of either their respective linear trends over the ESO period or their continuous piecewise linear trends with the breakpoint in winter 2003/04 (dashed lines in Fig. 2c,d), further supporting the scenario of oceanic regulation of the wintertime sea ice extent in the Barents Sea.

## Relation to Hemispheric Variability in the Sea Ice Cover

The remarkable decline of the Barents Sea ice cover since the mid-2000s has changed relationships between regional sea ice anomalies in the Northern Hemisphere^57^. The leading EOF mode of the winter mean SIC variability north of 40°N during the ESO period explains 27% of the SIC variance in this region. This mode mainly reflects the sea ice cover changes in the Barents Sea accompanied by weaker in-phase SIC variations in the Greenland Sea, and much weaker in-phase SIC variations in the Pacific sector (Fig. 3a, thin contours and color shading). Given the strong Barents Sea signal of this mode, its principal component time series (PC1_SIC−A40_) correlates highly with PC1_SIC−BS_ (*r* = 0.97; see Fig. 3c for comparison of the time series) and SIA_BS_ (*r* = −0.96; note that positive values of PC1_SIC−A40_ correspond to negative SIC anomalies in Fig. 3a). Therefore, PC1_SIC−A40_ reproduces all main features of the indices of SIC variability in the Barents Sea region, including the regime shift to low SICs in winter 2004/05 and record low SICs in the LAST3 winters. Given the tight relationship between the sea ice cover and SSTs in the Barents Sea region (Fig. 2), PC1_SIC−A40_ also reflects variations of SSTs in that region. It correlates remarkably highly (*r* = 0.98) with PC1_SST−BS_ (Fig. 2d, red curve).

A difference between the leading modes of the Arctic and Barents Sea SIC variability is in their association with SIC anomalies in the Greenland Sea, which is more significant for the Arctic mode (compare the patterns in Figs 3a and 1b). This difference should mainly reflect a change in the contribution of the Greenland Sea SICs to the total sea ice cover in the Barents/Nordic Seas region. In fact, unlike the Barents Sea, the Greenland Sea experienced most of its sea ice decline already during the EARLY period^58^ (see the thick black and blue lines in Fig. 2b for comparison of the mean location of the wintertime ice edge in the EARLY and LATE periods, respectively). This feature explains a slightly better (nearly perfect) agreement between PC1_SIC−A40_ and PC1_SIC−BS_ in the LATE period (*r* = 0.99), when SIC anomalies are small in the Greenland Sea but large in the Barents Sea, than in the EARLY period (*r* = 0.93). It is also consistent with a slightly stronger sea ice decline in the EARLY period associated with PC1_SIC−A40_ than PC1_SIC−BS_ (Fig. 3c, dashed lines) and with the selection of the winter 1997/98 as the onset time for the sea ice decline in PC1_SIC−A40_ (blue circle in Fig. 3c) by the objective OT detection method. Nevertheless, even in the case of PC1_SIC−A40_, most of the sea ice decline is due to the abrupt shifts in the LATE period.

## Relation to Pan-Arctic Variability in Air Temperature

The regime shifts of the Arctic climate system in the LATE period are also captured by the principal component time series (PC1_SAT−A70_) of the leading EOF mode of the winter mean SAT variability in the high Arctic north of 70°N (Fig. 3d, blue curve). The mode explains 48% of the wintertime SAT variance in the high Arctic and 92% of the corresponding variance in the SAT_nBS_ index defined as SATs averaged over the northern Barents Sea area (nBS box in Fig. 3b), with which PC1_SAT−A70_ correlates at *r* = 0.95 (see Fig. 3d for comparison of the time series). This tight relationship and the SAT anomaly pattern associated with PC1_SAT−A70_ (Fig. 3b) single out the Barents Sea region as the hotspot of climate change. In the anomaly pattern, a lobe of significant positive SAT anomalies, reflecting mainly the warming trend in the LATE period (significant at *p* = 0.01), covers the entire Arctic Ocean, but the largest anomalies appear just over the northern Barents Sea. PC1_SAT−A70_ correlates highly with PC1_SIC−A40_ (*r* = 0.93, *p* < 10^−3^) and indices of the sea ice cover and ocean temperature variability in the Barents Sea region (Table 2), portraying the Barents Sea hotspot as a feature of the coupled climate system.

**Table 2:** Cross-correlations between the principal component time series of selected first leading EOF modes of the winter mean Arctic surface climate variability and their correlations with concurrent area-averaged indices of this variability during the ESO period.

	PC1_SIC−BS_	PC1_SST−BS_	PC1_SIC−A40_	SST_sBS_	SAT_nBS_	SIA_BS_	SIA_BNS_
PC1_SIC−BS_	1	0.96	0.97	0.94	0.94	−0.99	−0.94
PC1_SST−BS_	0.96	1	0.98	0.96	0.91	−0.95	−0.92
PC1_SIC−A40_	0.97	0.98	1	0.94	0.92	−0.96	−0.94
PC1_SAT−A70_	0.90	0.92	0.93	0.85	0.95	−0.88	−0.84

The indices are defined in the caption to Table 1. All correlations are significant at the 99.9% or higher confidence level.

In both SAT indices (PC1_SAT−A70_ and SAT_nBS_), the onset time of the recent change obtained from the objective OT detection method is the same (winter 2003/04) as in all indices of the sea ice cover and SST variability in the Barents Sea region. When the method is applied to smoothed time series (11-yr running mean data), the onset time appears simultaneously (in winter 2000/01) in all SST and sea ice cover indices, including PC1_SIC−A40_ (see column OT_11_ in Table 1). However, in the smoothed SAT indices, the onset time appears somewhat earlier (in the mid-1990s). While this discrepancy may result from uncertainties inherent in the atmospheric reanalysis from which the SAT data are derived, it could also reflect an influence of sea ice cover anomalies in the Pacific sector of the Arctic Ocean on pan-Arctic SAT variations. Such a possibility is suggested by an earlier onset time of the sea ice decline in that sector (in the early 1990s) compared to the Atlantic sector^54^.

## Relation to Summertime Subsurface Ocean Temperature Anomalies

In the Atlantic domain of the shallow Barents Sea, the warm and salty AW extends nearly throughout the entire water column and is ventilated via air-sea interactions during the cooling season when most of the heat advected with AW to the Barents Sea is lost to the atmosphere^59^. In the Arctic Ocean, the relatively warm and salty AW layer is submerged below the cold and fresh surface mixed layer, from which AW is separated by a highly stratified cold halocline^60^. In order to analyse the spatiotemporal structure of the subsurface ocean heat content in the Barents/Nordic Seas region, June-August (JJA) mean gridded fields of the vertically-averaged temperature in the 150–250 m depth layer (*T*_150−250_) are constructed from scattered observations (see Methods). The 150–250 m depth range corresponds to the warmest, uppermost part of the AW layer below the Arctic Ocean halocline near the northern Barents Sea boundary^48^ and encompasses the AW salinity maximum in the open water at the entrance to the Barents Sea^61^.

All major features of the EARLY period climatology of *T*_150−250_ also appear in the corresponding LATE period climatology, although the subsurface waters are generally warmer in the LATE period (compare the upper panels in Fig. 4 and note areas of missing data marked by dark shading of the grid cells). The strongest warming is found in the Barents Sea, where the difference between the LATE and EARLY means of *T*_150−250_ reaches about 1.5 °C (Fig. 4c). A notable warming by about 1 °C also appears in the West Spitsbergen Current, in the AW pathway to the Arctic Ocean north of Svalbard and the AW recirculation, westward in Fram Strait and then southward in the East Greenland Current along the East Greenland shelf slope (see the thin contours in Fig. 4a–e for the 300 and 1000 m isobaths).

To demonstrate a strong dependence of the sea ice cover on subsurface ocean heat anomalies in the Barents/Nordic Seas region, Fig. 4d and 4e show patterns of the difference in the composite mean of *T*_150−250_ between the summers preceding the winters with prevailing “open water” (ICE^−^) conditions and the summers preceding the winters with prevailing “icy” (ICE^+^) conditions during the EARLY and LATE periods, respectively. The selection of the ICE^−^ and ICE^+^ winters (see Methods for details) is based on the SIA_BNS_ index defined as the sea ice area over the Barents and Nordic Seas (BNS box in Fig. 4d). During the EARLY period, ocean temperatures higher by up to about 1 °C in the ICE^−^ years than the ICE^+^ years are found in the West Spitsbergen Current, north of Svalbard, in the East Greenland Current, as well as in the western Barents Sea (Fig. 4d). During the LATE period, the data coverage is less extensive, but the available data again show positive temperature differences between the ICE^−^ and ICE^+^ years in the western Barents Sea, Fram Strait and the East Greenland Current (Fig. 4e).

To statistically quantify the relationship between the changing sea ice cover and subsurface ocean heat, a time series of summer mean AWT anomalies (AWT_SSS_ index) is constructed (see Methods) using temperature profiles in the southern Svalbard slope area (SSS box in Fig. 4e). The AWT_SSS_ index (Fig. 4f, blue curve) exhibits nonsignificant warming through the EARLY period (*p* > 0.1) and significant warming through the LATE period (*p* = 0.03). It correlates highly with the following winter SIA_BNS_ index (*r* = −0.92; see Fig. 4f for comparison of the time series) and, consequently, with the PC of the leading EOF mode of the wintertime hemispheric SIC variability (*r* = 0.90; see column *r*_AWT_ in Table 1 for the correlations of AWT_SSS_ with this and other indices). These high correlations and the correspondence between the onset time of the recent changes, identified by the objective OT detection method in winter 1997/98 for SIA_BNS_ and the preceding summer for AWT_SSS_ (Fig. 4f, red and blue circles), indicate that ocean heat anomalies influence the sea ice cover in both the Barents Sea and the Greenland Sea. However, as shown below, the influence on the Greenland Sea SICs is limited to the EARLY period.

## Lagged Relationships in the EARLY and LATE Periods

**Figure 5:**
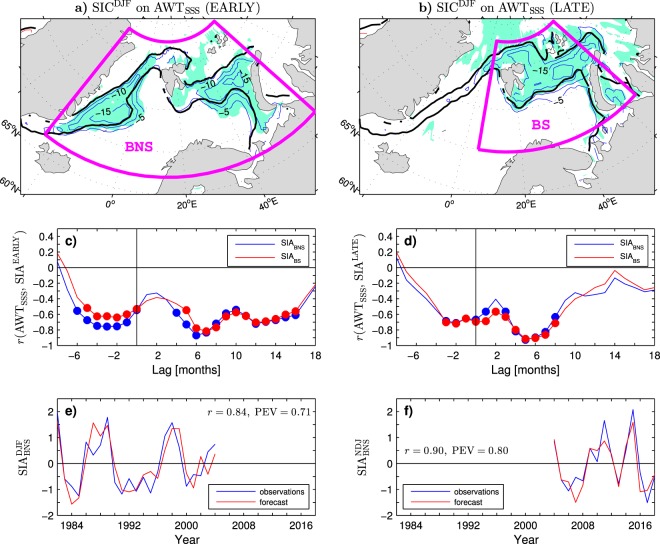
Spatial structure, seasonal evolution and predictability of the sea ice concentration anomalies in the Barents/Nordic Seas region during the EARLY and LATE periods. (**a**,**b**) (thin contours and colour shading) Detrended winter mean SIC anomalies regressed onto the detrended previous summer anomalies of Atlantic water temperature (AWT_SSS_ index, blue curve in Fig. 4f) in the EARLY (**a**) and LATE (**b**) periods. The thin contour and shading colours are explained in the caption to Fig. 1. The CI is 5% per unit AWT_SSS_ index re-standardised for the anomalies in the two periods. The thick black lines (15% and 85% SIC contours) delineate the marginal ice zone in the two periods. The maps were generated by MathWorks MATLAB R2014a with M_Map (http://www.eoas.ubc.ca/rich/map.html). (**c**,**d**) Time-lagged correlation coefficient of the summer mean AWT_SSS_ anomalies with the seasonal (3-month) mean anomalies of the sea ice area in (blue line) the Barents/Nordic Seas (BNS box in **a**) and Barents Sea (BS box in **b**) regions during the EARLY (**c**) and LATE (**d**) periods. The filled circles denote correlations statistically significant at the 95% confidence level. Positive (resp. negative) lags correspond to the AWT_SSS_ anomalies leading (resp. lagging) the SIA anomalies. (**e**,**f**) Time series of the observed (blue curve) and predicted (red curve) wintertime SIA anomalies in the Barents/Nordic Seas region in the EARLY (**e**) and LATE (**f**) periods. The predictions are for the DJF and NDJ mean SIA_BNS_ anomalies in the EARLY and LATE periods, respectively. They are based on leave-1-yr-out cross-validation forecasts with the previous JJA mean AWT_SSS_ anomalies as the predictor. Each year on the horizontal axis includes January of the winter season.

The pattern of the wintertime SIC anomalies associated with the previous summer ocean heat anomalies (AWT_SSS_ index) differs considerably between the EARLY and LATE periods (see Fig. 5a,b for the regression of detrended data). In the EARLY period, significant SIC anomalies appear all along the marginal ice zone of the Nordic Seas as well in the Barents Sea (see Fig. 5a; thin contours and colour shading for the SIC anomalies and thick black lines for the climatological 15% and 85% SIC contours). In the LATE period, significant SIC anomalies are found only in the northward shifted marginal ice zone of the Barents Sea region (Fig. 5b). In that region, in the EARLY period, large local SIC anomalies (above 15% per unit AWT_SSS_ index) appear only in the northeastern Barents Sea while in the LATE period such anomalies extend across the entire northern Barents Sea. In addition, there are differences in the seasonal timing of the strongest sea ice response to oceanic forcing, as indicated by the time-lagged correlations of the summer AWT_SSS_ anomalies with the seasonal (3-month running) mean anomalies of the sea ice cover over the Barents/Nordic Seas region (SIA_BNS_ index) and the Barents Sea alone (SIA_BS_ index) in the EARLY and LATE periods (Fig. 5c,d). In the EARLY period, the SIA_BNS_ anomalies reach a maximum postsummer correlation (*r* = −0.87) in winter at lag +6 months (Fig. 5c, blue curve). For the SIA_BS_ anomalies, the maximum postsummer correlation is slightly lower (*r* = −0.82) and occurs slightly later, in late winter (January-March, JFM) at lag +7 months (Fig. 5c, red curve). In the LATE period, the maximum postsummer correlation is higher (*r* = −0.93 for SIA_BNS_ and *r* = −0.92 for SIA_BS_) and occurs already in early winter (November-January, NDJ) at lag +5 months (Fig. 5d). Similar relationships hold for the raw (undetrended) data.

To test the statistical significance of the recent changes in the relationship between the wintertime surface climate indices and the previous summer subsurface ocean temperature in the Barents Sea region, a Monte Carlo method is employed (see Methods). Empirical *p*-values for the tested difference in the correlation coefficients of all analysed raw winter mean time series with the previous summer AWT_SSS_ index between the EARLY and LATE periods are included in Table 1 (column *p*_Δ*r*_). The increase of the correlation in the LATE period is evident for all indices of the variability in the Barents Sea region. It is significant (*p*_Δ*r*_ between 10^−3^ and 10^−2^) for the area-averaged SICs in the Barents Sea, the PC1s of the SIC and SST variability in that region, and area-averaged SATs in the northern Barents Sea area. The increase is most significant (*p*_Δ*r*_ ≈ 10^−4^) for the area-averaged SSTs in the southern Barents Sea. This increase suggests that the enhanced impact of summertime anomalies of the subsurface ocean temperature on the wintertime climate variability in the Barents Sea region reflects their stronger control of postsummer SSTs on the open water side of the sea ice edge (see the next section but one for possible mechanisms).

## Predictability of the Wintertime Surface Climate Variability

In order to demonstrate the predictive value of the lagged relations identified above, empirical forecast models are constructed for the winter and early winter SIA_BNS_ in the EARLY and LATE periods, respectively. The forecast models are based on the linear regression with the leave-1-yr-out cross-validation (see Methods) and use the summertime AWT_SSS_ index as the predictor. The standardised time series of the predicted and observed interannual (detrended) SIA_BNS_ anomalies are compared in Fig. 5e,f. High values of the correlation skill score (CSS) and the proportion of explained variance (PEV) of the forecasts indicate that the sea ice cover anomalies in the Barents/Nordic Seas region can be predicted with high confidence. Consistent with the correlations analysed above, the skill scores of the forecast of the SIA_BNS_ anomalies are slightly higher in the LATE period (CSS = 0.90 and PEV = 0.80) than the EARLY period (CSS = 0.84 and PEV = 0.71). Similarly, the skill scores of the forecast of the raw SIA_BNS_ index from the undetrended AWT_SSS_ anomalies are higher in the LATE period (CSS = 0.94 and PEV = 0.88) than the EARLY period (CSS = 0.82 and PEV = 0.67).

The increase of predictability is more pronounced for the sea ice cover in the Barents Sea alone. For instance, the PEV score for the early winter SIA_BS_ in the LATE period (0.87) is higher by about 0.35 than the PEV score for the late winter SIA_BS_ in the EARLY period (see Table 3 for the skill scores of the forecasts of the raw early and late winter area-averaged variables in the Barents Sea region). Given the strong linkage of the wintertime SICs in the Barents Sea region to the concurrent SSTs in that region (Fig. 2), high skill scores are also obtained for the prediction of these SSTs. The scores are again higher in the LATE period (PEV = 0.84 for the early winter SST_sBS_ index) than the EARLY period (PEV = 0.40 for the late winter SST_sBS_ index). The most remarkable increase in predictability is observed for the atmospheric hotspot over the northern Barents Sea where the SATs were not predictable in the EARLY period but to a large extent predictable in the LATE period (PEV = 0.70 for the early winter SAT_nBS_ index).

**Table 3:** Correlation skill score (CSS) and the proportion of explained variance (PEV) in leave-1-yr-out cross-validation forecasts of undetrended wintertime surface climate variables in the Barents Sea region from the undetrended preceding summer (JJA) Atlantic water temperature in the southern Svalbard slope area (SSS box in Fig. 4e) for the EARLY (winters 1981/82–2003/04) and LATE (winters 2003/04–2017/18) subperiods of the ESO period.

Period	Season	SIA_BS_		SST_sBS_		SAT_nBS_	
		CSS	PEV	CSS	PEV	CSS	PEV
EARLY	NDJ	0.38	0.11	0.35	0.08	−0.05	−0.12
EARLY	JFM	**0.71**	0.51	**0.63**	0.40	0.24	0.03
LATE	NDJ	**0.93**	0.87	**0.92**	0.84	**0.84**	0.70
LATE	JFM	**0.88**	0.78	**0.84**	0.71	**0.62**	0.37

The predictands are the SIA in the Barents Sea region (BS box in Fig. 1b), SST in the southern Barents Sea (averaged over the sBS box in Fig. 2a), and SAT in the northern Barents Sea area (averaged over the nBS box in Fig. 3b). The forecasts are for the early winter (NDJ) and late winter (JFM) seasons. The CSS values significant at the 99% (99.99%) confidence level are in boldface (boldface and italic).

## Possible Mechanisms

**Figure 6:**
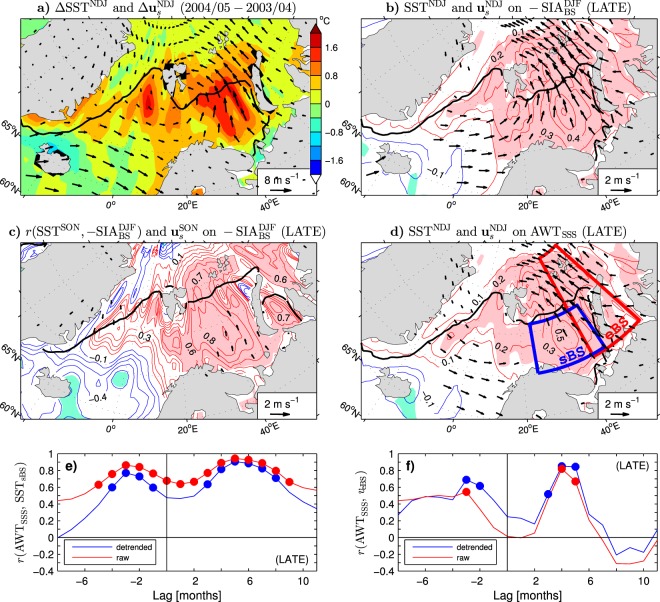
Spatial structure and seasonal evolution of SST and surface wind anomalies in the Barents/Nordic Seas region during the LATE period. (**a**) Difference in early winter (NDJ) SST (colours) and **u**_*s*_ (arrows) between 2004/05 and 2003/04. (**b**) Early winter anomalies of SST and **u**_*s*_ regressed onto the winter (DJF) SIA_BS_ index multiplied by −1. (**c**) Correlation coefficient of the autumn (SON) SST anomalies with the following winter SIA_BS_ index (multiplied by −1) and autumn **u**_*s*_ anomalies regressed onto that index. (**d**) Early winter anomalies of SST and **u**_*s*_ regressed onto the previous summer AWT_SSS_ index. (**e**) Time-lagged correlation coefficient of the detrended (blue curve) and raw (red curve) summer AWT_SSS_ index with the corresponding seasonal mean SSTs averaged over the southern Barents Sea (sBS box in **d**). (**f**) As in (**e**) but for the seasonal mean surface meridional winds (positive northward) averaged over the eastern Barents Sea (eBS box in **d**). In (**b**–**d**) the time series were detrended before the analysis. The thin contour and shading colours (explained in the caption to Fig. 1) are for the SST anomalies. The CI is 0.1 °C per unit SIA_BS_ index, 0.1 and 0.1 °C per unit AWT_SSS_ index, respectively. The anomalies of **u**_*s*_ are subsampled and masked if both components are nonsignificant at the 95% confidence level. In (**a**–**d**) the thick black lines are the mean 15% SIC contours in early winter 2004/05, early winters and autumns of the LATE period, and early winters of the LATE period, respectively. The maps were generated by MathWorks MATLAB R2014a with M_Map (http://www.eoas.ubc.ca/rich/map.html). In (**e**,**f**) the filled circles denote correlations statistically significant at the 95% confidence level. Positive (resp. negative) lags correspond to AWT_SSS_ leading (resp. lagging) the surface variables.

We have demonstrated that variations of the wintertime sea ice cover in the Barents Sea are strongly coupled to the concurrent SST anomalies on the open water side of the ice edge (Fig. 2) as well as the previous summer subsurface ocean heat anomalies (Fig. 4, Table 1), especially in the LATE period (Fig. 5). These relations and the high predictability of the wintertime SSTs in the southern Barents Sea from the AWT_SSS_ index (Table 3) are consistent with the scenario that the sea ice cover in the Barents Sea is regulated by subsurface ocean heat anomalies that, in summer, are insulated from interactions with the atmosphere by a shallow seasonal pycnocline, but during the cooling season are entrained into the deepening ocean surface mixed layer and subsequently affect sea ice formation^49,50^. This scenario is further supported in Fig. 6, which emphasises the coupled system variability in the LATE period. In particular, it demonstrates that the abrupt sea ice decline between the EARLY and LATE periods (Fig. 1c,d) was associated with a warm SST pulse in the open water (see colours in Fig. 6a for the SST difference between early winters 2004/05 and 2003/04), as was the event of spectacularly light ice conditions during the LAST3 winters (Fig. 2a,c). It also shows a close correspondence between patterns of the interannual SST anomalies in early winter associated with the one month later (winter) anomalies of SIA_BS_ and the previous summer anomalies of AWT_SSS_ (see thin contours and colour shading in Fig. 6b,d, respectively, and note that the sign of the SIA_BS_-covariant SST anomalies in Fig. 6b corresponds to light ice conditions). The strong dependence of the wintertime sea ice cover on ocean heat anomalies emerging at the surface at the onset of the cooling season is illustrated by the pattern of correlations between the SST anomalies in autumn (September-November, SON) and the following winter anomalies of SIA_BS_ (see Fig. 6c, thin contours and colour shading, and note the reversed sign of the correlations). In the central Barents Sea, these correlations reach values of up to 0.91. This relationship mainly reflects the sea ice response to reemerging SST anomalies, as indicated by the evolution of time-lagged correlations between the seasonal mean SSTs averaged over the southern Barents Sea (SST_sBS_ index) and the summer AWT_SSS_ index, shown in Fig. 6e for both the interannual anomalies (blue curve) and raw data (red curve). These correlations exhibit a seasonal cycle with lowered values in summer and peaks in the postsummer and presummer seasons. Maximum presummer correlations (*r* = 0.77 for the interannual anomalies and *r* = 0.86 for the raw data) occur in spring (lag −3 months), and maximum postsummer correlations (*r* = 0.91 for the interannual anomalies and *r* = 0.94 for the raw data) are found in early winter (lag +5 months).

While the wintertime sea ice cover in the Barents Sea is coupled to ocean heat anomalies, it should also respond to anomalous local winds^52,53,62^. In the LATE period, this response is suggested by the pattern of the difference in the surface wind vector (**u**_*s*_) between early winters 2004/05 and 2003/04 (arrows in Fig. 6a) and the pattern of the interannual anomalies of **u**_*s*_ in early winter associated with the one month later anomalies of SIA_BS_ (shown in Fig. 6b as arrows with reversed direction and masked if both wind components are nonsignificant at the 95% confidence level). Both patterns exhibit an anomalous cyclone around Svalbard and southerly wind anomalies across the entire Barents Sea corresponding to light ice conditions. In the marginal ice zone, warm air advection by southerly wind anomalies should amplify atmospheric warming resulting from upward heat flux anomalies induced by the reduced sea ice cover. Over the open water, it should reduce ocean heat loss to the atmosphere, generate or amplify warm SST anomalies, and consequently inhibit sea ice formation^62,63^. In addition, southerly wind anomalies should reduce sea ice advection from the nearby Kara Sea and the Arctic Ocean^13,54^. Moreover, variations of oceanic circulation in the Barents Sea during the cold season are mainly wind-driven^64^. Therefore, the anomalous winds may also contribute to the SST/SIC variability via changes in heat transport by the wind-driven currents^44,65^.

A key point here is that the wind anomalies that influence the wintertime sea ice cover in the Barents Sea are not independent of the SST anomalies but, in the first place, represent a response to sea ice cover anomalies driven by ocean heat variations. Consistent with this scenario, in autumn, when the SSTs in the Barents Sea open water associated with the following winter SIA_BS_ anomalies are highly significant, the corresponding wind anomalies are barely significant at a few points (arrows in Fig. 6c). The response of atmospheric circulation to ocean heat variations is further corroborated by the pattern of significant early winter wind anomalies associated with the previous summer AWT_SSS_ anomalies (arrows in Fig. 6d), which closely resembles the corresponding pattern of the SIA_BS_-covariant wind anomalies (Fig. 6b). The wind response is strong, as indicated by the evolution of time-lagged correlations between the seasonal mean *v*_eBS_ index defined as the meridional component of **u**_*s*_ (positive northward) averaged over the eastern Barents Sea (eBS box in Fig. 6d) with the summer AWT_SSS_ index. For the interannual anomalies (see Fig. 6f, blue curve), significant postsummer correlations arise in autumn and reach a maximum (*r* = 0.85) in late autumn (lag +4 months) and early winter. For the raw data (see Fig. 6f, red curve), the maximum postsummer correlation is nearly as large (*r* = 0.82 in late autumn) as for the interannual anomalies. The postsummer AWT_SSS_-covariant anomalies of *v*_eBS_ become nonsignificant already in winter (lag +6 months), while the corresponding SST anomalies remain significant up to the following spring (lag +9 months; see Fig. 6e) and maintain significant sea ice cover anomalies to the end of the sea ice growth season in the early spring (lag +8 months; see Fig. 5d).

## Discussion

Over recent decades, the insulation effect of the cold halocline has gradually weakened along the cyclonic AW pathway around the Arctic Ocean probably through a chain of positive feedbacks between the declining sea ice cover, weakening of the halocline stratification, increasing vertical mixing, and growing upward heat fluxes from the shallowing AW layer^12,48^. These feedbacks could have become particularly effective since the mid-2000s in the northern Barents Sea where the highly stratified Arctic domain meets the weakly stratified Atlantic domain^13^ and, consequently, accelerated Arctic amplification. It was proposed that a key role in these feedbacks is played by declining sea ice import to the Barents Sea from the Kara Sea and the associated loss of freshwater leading to weakened ocean stratification^13^. While this process may indeed contribute to climate feedbacks in the Barents Sea hotspot area, the present study underscores ocean heat anomalies as a flywheel of the recent coupled climate changes and variability in this area. Increased or warmed inflows of AW inhibit sea ice growth leading to enlargement of the Atlantic domain and the attendant northeastward recession of the wintertime sea ice edge in the Barents Sea^44,49,50,65^. The enhanced surface heat loss to the atmosphere over the increased open water area during the ice-growth season leads to tropospheric warming^66^. This warming may be amplified by positive feedback between the sea ice decline, induced anomalous atmospheric circulation and consequent changes in ocean heat transport by the wind-driven currents. A climate modelling study shows that such feedback might have been responsible for the early 20th-century warming in the Arctic^67^. Some conceptual models suggest that such feedback may also drive quasi-decadal oscillations in the Arctic climate system^68,69^. As proposed in an earlier study^62^ and supported here using longer time series, a key role in such feedback could be played by subsurface ocean heat anomalies that emerge or reemerge on the surface at the ice edge during the autumn-to-winter ventilation of the ocean and subsequently regulate the sea ice advance and local winds. However, if and how changes in heat transport due to wind-driven current anomalies have contributed to the recent climate feedbacks in the Barents Sea region remains to be demonstrated by numerical models.

Oceanic forcing is of paramount importance for seasonal sea ice prediction. The statistical analysis and empirical forecast experiments reported here indicate that the high predictability of the wintertime sea ice cover in the Barents Sea region from earlier subsurface ocean temperature anomalies^49,50^ did not only survive through the climate shift of the mid-2000s but has even increased since then. The greater oceanic control of the wintertime sea ice extent in the Barents Sea may have implications not only for local climate feedbacks and marine ecosystems but also for Arctic/mid-latitude linkages. These linkages are manifested in, for instance, anomalously cold Eurasian winters when Arctic temperatures rise (Fig. 3b,d) - a phenomenon subject to intense recent research^16–18,22,70–73^.

## Methods

### Datasets and indices of surface climate variability

Seasonal mean fields of SST and SIC are constructed from the NOAA Optimum Interpolation Sea Surface Temperature Version 2 monthly mean fields derived from remote and *in situ* observations^74^ and provided on a 1° latitude ×1° longitude grid for the period since December 1981 to present (https://www.esrl.noaa.gov/psd/). Seasonal mean fields of SAT and surface wind vector (**u**_*s*_) are computed from the monthly mean fields of air temperature at the 2-m height and wind at the 10-m height, respectively, derived from the NCEP/NCAR reanalysis^75^ and provided on a gaussian grid of approximately 1.8-degree resolution (https://www.esrl.noaa.gov/psd/).

Basic indices of regional surface climate variability are obtained by averaging of the seasonal mean SIC, SST and SAT fields over the Barents/Nordic Seas (67°–83°N, 25°W–65°E), Barents Sea (70°–83°N, 5°–65°E), southern Barents Sea (70°–76°N, 25°–50°E) and northern Barents Sea (78°–82°N, 25°–70°E) domains. The averaged SIC (fraction of the ocean surface covered by sea ice) multiplied by the total area of the domain of interest is referred to as the sea ice area (SIA). While the focus is on the winter (December-February, DJF) season, some indices are also computed for other seasons in order to explore time-lagged relationships.

Principal component (PC) time series of the EOF decomposition of wintertime field anomalies^76^ serve as supplementary indices of variability. The field anomalies are obtained by subtracting local mean values from the raw data. In order to account for different areas represented at each grid point, the anomalies are weighted by the square root of the cosine of latitude prior to the EOF decomposition. Only the first leading modes that account for the largest fraction of the total variance in the data are analysed. The modes are computed for the SIC and SST anomalies over the Barents Sea region (BS box in Figs 1b and 2b), SIC anomalies over the Northern Hemisphere north of 40°N, and SAT anomalies in the high Arctic north of 70°N. All these modes are well separated from the corresponding second modes according to North’s “rule of thumb”^77^. Their PCs are denoted as PC1_SIC−BS_, PC1_SST−BS_, PC1_SIC−A40_, and PC1_SAT−A70_, respectively. The cross-correlations of these PCs and their correlations with other indices of wintertime climate variability are given in Table 2.

### Subsurface ocean datasets and their processing

In order to construct fields of subsurface ocean temperature anomalies, data from three sources are used. The main database (UDASH) is a unified collection of hydrographic observations for the Arctic Ocean and subarctic seas north of 65°N for the period 1980–2015 compiled by the Alfred Wegener Institute, Bremerhaven, Germany^55^. It consists of thoroughly quality-checked profiles of temperature and salinity measured mainly with conductivity/temperature/depth probes, bottles, mechanical thermographs and expandable thermographs from all publicly available data sources, including the Oceanographic Database of the International Council for the Exploration of the Sea (ICES)^78^. Additional hydrographic data from the ICES from 2016 and 2017 are used to extend the UDASH archive by two years. The UDASH/ICES archive is filtered to retain only summertime (June-August, JJA) temperature profiles and augmented with temperature profiles from the Arctic Experiment (AREX) database of the Institute of Oceanology, Sopot, Poland. The latter compiles measurements from summertime cruises of R/V *Oceania* in the West Spitsbergen Current area since the late 1980s^46,79,80^. The final dataset spans all summers from 1981 to 2017.

All summertime temperature profiles are vertically averaged over the layer between selected depth levels and then gridded. Stations not spanning the entire layer are disregarded, as are repeated stations. The vertically-averaged temperature *T*_*i*_ at each hydrographic station *s*_*i*_ is then corrected for the seasonal cycle using the monthly mean temperature data from the University of Washington Polar Science Center Hydrographic Climatology 1.0 (PHC)^81^, which are vertically-averaged and interpolated onto the location of *s*_*i*_. Each *T*_*i*_ is referenced to the middle of the season of interest. Climatological averages of the summer means of the vertically-averaged temperature are then constructed for the EARLY (summers 1981–2003) and LATE (summers 2004–2017) subperiods. Anomalies are computed for all summers, and their composite means are constructed for selected sets of years. To this end, first, the local average *T*_*l*_ is computed at the location of each station. The average at the given station *s*_*l*_ is calculated using data from all stations inside a circular domain with the radius *R*_0_ around *s*_*l*_ except for the stations, if present, classified as outliers (those with the temperature differing from *T*_*l*_ by at least five standard deviations). If no station is found inside the search area, station *s*_*l*_ is eliminated. The distance of half a degree of latitude (about 56 km) is selected as *R*_0_.

The gridded fields of the vertically-averaged temperature are computed with a resolution of 0.5° in latitude and 1.5° in longitude. The climatological mean values are calculated by weighted averaging of the local averages *T*_*l*_ at stations *s*_*l*_ found inside a circular domain of influence with the radius *R*_0_ around the centre of the given grid cell. Local inverse distance weighting is employed with weights *w*_*l*_ = (*R*_0_^2^−*d*_*l*_^2^)/(*R*_0_^2^ + *d*_*l*_^2^), where *d*_*l*_ is the distance of station *s*_*l*_ from the cell centre. The weights are normalised so that they sum to one. The *T*_*l*_ outliers (classified as such based on the 5-standard-deviation criterion applied to the *T*_*l*_ data within the search area) are excluded from the computation of the climatological means. The means are calculated only for the grid cells with at least two stations in the search area. The gridded temperature anomalies are constructed using the same spatial averaging procedure but applied, for each summer, to the departures *T*_*a*_ of the vertically-averaged temperatures *T*_*i*_ from the corresponding local climatological means *T*_*l*_ in the EARLY period. This procedure is a modified version of the method applied earlier to construct temperature anomalies from scattered hydrographic observations in the North Atlantic^82^.

A reliable time series of the summer mean AWT anomalies (AWT_SSS_ index) is constructed using temperature data averaged over the 100–300 m depth layer in the southern Svalbard slope (SSS) area of the western Barents Sea opening (73°–75°N, 13°–20°E). First, the local anomalies of the vertically-averaged temperature from its EARLY period climatology are computed for all stations inside the SSS area. These anomalies are supplemented with the local anomalies at two additional stations per each summer, by one at (or closest to) the southern and northern limits of the SSS area. Overall, a set of 1601 local anomalies is used. The set has a very irregular time distribution but includes at least ten data in each summer. Then, for each summer, the local anomalies are interpolated and averaged following the procedure outlined in an earlier study^63^. The resulting time series of the AWT_SSS_ anomalies are standardised by subtracting their mean value (0.25 °C) and dividing by their standard deviation (0.6 °C). Its correlations with all analysed indices of climate variability in the following winter are included in Table 1 (column *r*_AWT_).

### Regime change detection

An automatic calculation of regime shifts in the analysed indices of climate variability is carried out using the method devised in an earlier study^56^. First, the cut-off length *l* of the regimes to be determined for a given index *I* is set. Then, the difference between mean values of two subsequent regimes that would be statistically significant according to the Student’s *t*-test at the probability level *p* = 0.05 is computed. Next, the mean of the initial *l* values of *I* is set as an estimate of the current regime, and the range of values that should be exceeded in the subsequent *l* years to qualify for a new regime is calculated. If the difference between the current regime estimate and the next datum falls outside this range, the year *j* of that datum is considered as a potential start point of the first new regime. If not, the estimate of the current regime is updated using that datum and the preceding *l* − 1 data. The procedure is repeated until the potential start point of the first new regime is established. The confidence of a regime shift in year *j* is tested using the regime shift index based on data in that year and subsequent *l* − 1 years if available^56^. If the test fails, the search for the potential start point of the first new regime is continued. After this point (referred to as CP1 in the main text) is found, the whole procedure is repeated to find the start point of the second (CP2) and next new regimes, if present. The results of the search for the CP points using 11 years as the cut-off length *l* are reported in Table 1. The results are found to be insensitive to small changes in *l*.

As the above method of regime change detection does not detect early onset times of nonlinear but initially gradual transitions to new regimes, a version of the method for objective detection of such transitions employed in an earlier study^54^ is also used here. First, the beginning and end points of a given time series are set as base points of the analysis. Next, the orthogonal distance between each datum and the straight line joining the base points is calculated, and the year *j* of the maximum distance is found. If the maximum distance is greater than a threshold *ε*, year *j* is marked as a potential onset time of a new regime (referred to as OT in the main text). The time series is then split at year *j* into two segments, and other potential OTs are sought, by one in each segment. The procedure is applied recursively to all segments resulting from splitting each segment from the previous iteration until the orthogonal distance to the line joining the base points of the current segment is less than or equal to *ε*. The value of *ε* is set to 1.5 of the standard deviation of the original time series. From all potential OTs, the one which yields a minimum root mean square error (RMSE) of fitting the initial time series to the piecewise linear trend with the breakpoint at the given potential OT is selected as the final, single OT. If two or more potential OTs yield an equal (to within 0.5%) RMSE, the one is retained for which the onward linear trend is the largest. The same procedure is applied to obtain estimates of the onset time, denoted as OT_11_, based on the 11-yr running mean of the time series. The OT and OT_11_ points for all analysed indices of climate variability are reported in Table 1.

### Statistical analyses

Correlation analysis is carried out to quantify relationships between different indices of climate variability and between these indices and the regressed fields. The statistical significance of the correlation coefficient *r* is estimated using a two-sided Student’s *t*-test. The serial correlation in the time series is taken into account by employing an effective sample size defined as *N*_*eff*_ = *N*_0_(1 − *r*_*a*_*r*_*b*_)/(1 + *r*_*a*_*r*_*b*_), where *N*_0_ is the length of the series while *r*_*a*_ and *r*_*b*_ are the lag-one autocorrelations of the correlated series *a* and *b*^83^. The statistical significance of linear trends is estimated with a one-tailed Students’s *t*-test. The effective sample size for this test is obtained using the lag-one autocorrelation of the regression residuals^84^. The 95% confidence level is used as the threshold for marking significant anomalies in all regression maps and maps of differences between composite means. The statistical significance of the composite mean differences is tested using the standard two-sample *t*-test^76^.

Composite mean differences of the subsurface ocean temperature are calculated only for the grid cells for which data from at least two summers contribute to the mean in each of the epochs being compared. These differences are computed between the LATE and EARLY subperiods and, in these subperiods, between summers preceding light ice (ICE^−^) and heavy ice (ICE^+^) winters. The ICE^−^ and ICE^+^ winters are selected using the SIA_BNS_ index (red curve in Fig. 4f). Winters in which the magnitude of the anomaly of SIA_BNS_ from its mean value in the given period is not smaller than a threshold value (0.75 of the standard deviation in that period) are retained. In the EARLY period, this criterion yields six ICE^−^ winters (1983/84, 1984/85, 1990/91, 1992/93, 1994/95, 1999/2000) and six ICE^+^ winters (1981/82, 1985/86, 1988/89, 1996/97, 1997/98, 2003/04). In the LATE period, the LAST3 winters (2015/16–2017/18) of the extremely low ice cover are selected as the ICE^−^ winters and four winters (2008/09–2010/11 and 2014/15) with the highest values of SIA_BNS_ after 2003/04 are selected as the ICE^+^ winters.

Monte Carlo simulations are carried out to test whether the recent increase of correlations between indices of wintertime climate variability and the preceding summer AWT_SSS_ index is statistically significant. As a first step, *N* (set to 10^4^) synthetic realizations of 23 pairs of data of the AWT_SSS_ index and a given wintertime climate index *I* are generated by randomly subsampling the data from the EARLY period. Then, the number *n* of the realizations for which the correlation between the synthetic AWT_SSS_ and *I* indices exceeds or is equal to the correlation between the observed 15-year-long time series of these indices in the LATE period is found. The ratio *n*/*N* yields an estimate of the *p*-value from the Monte Carlo test for the difference between correlations in the EARLY and LATE periods. This estimate for all analysed wintertime variables is included in Table 1 (column *p*_Δ*r*_).

### Empirical forecasts

Forecast models are constructed for the wintertime sea ice area over the Barents/Nordic Seas region (SIA_BNS_ index) in the EARLY and LATE subperiods of the ESO period. The summertime Atlantic water temperature in the southern Svalbard slope area (AWT_SSS_ index) is used as the predictor. The predictor and predictand time series are either undetrended or linearly detrended over the forecast period. Additional forecast experiments are carried out for the undetrended sea ice cover in the Barents Sea alone (SIA_BS_ index), area-averaged SSTs in its southern part (SST_sBS_ index), and area-averaged SATs in its northern part (SAT_nBS_ index). The forecasts are based on the standard linear regression method with the leave-1-yr-out cross-validation scheme^85^. In the leave-1-yr-out experiments, the training data are strictly separated from the testing data. One year is first excluded from *K* years of data. The forecast model is then trained on the data from the remaining years and tested on the excluded data. The procedure is repeated for each year, providing *K* test results from which two forecast skill scores are estimated. The first one is the correlation skill score (CSS) defined as the correlation coefficient between the forecasts *F* (predicted values) and their targets *T* (predictand values). The second one is the proportion of explained variance (PEV) defined as P*EV* = 1 − *σ*^2^(*F* − *T*)/*σ*^2^(*T*), where *σ*^2^ stands for the variance^76^. PEV is positive if the regression model is better than the climate reference model (*F* = 0 in our case). It becomes unity for a perfect model, that is, when *σ*^2^(*F* − *T*) = 0, and minus unity for a random forecast.
