# Author Correction: Subsurface ocean flywheel of coupled climate variability in the Barents Sea hotspot of global warming

**DOI:** 10.1038/s41598-020-61544-8

**Published:** 2020-03-10

**Authors:** Pawel Schlichtholz

**Affiliations:** Institute of Oceanology, Polish Academy of Sciences, Powstancow Warszawy 55, 81-712 Sopot, Poland

Correction to: *Scientific Reports* 10.1038/s41598-019-49965-6, published online 23 September 2019

This Article contains errors.

Figure 1b incorrectly shows SIC anomalies over the winters 2002/03-2016/17 instead of the winters 1981/82-2017/18. The correct Figure 1 appears below.

**Figure 1:**
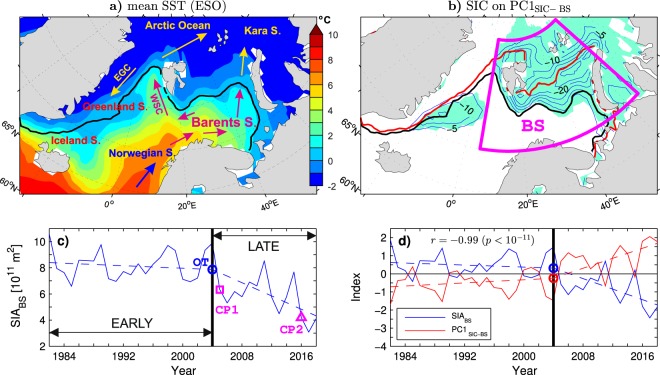
.

In Figure 3b, the geographical limits of the northern Barents Sea (nBS) area are incorrectly marked. The correct Figure 3 appears below as Figure 2.

**Figure 2:**
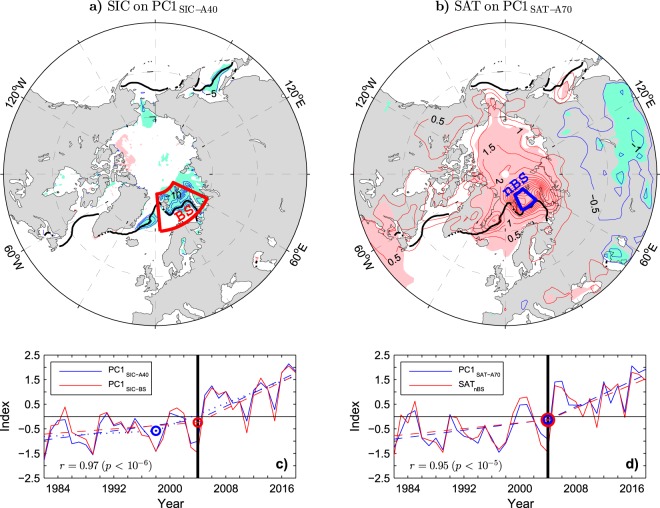
.

In Figure 4f, the inscription is incorrect. The correct Figure 4 appears below as Figure 3.

**Figure 3:**
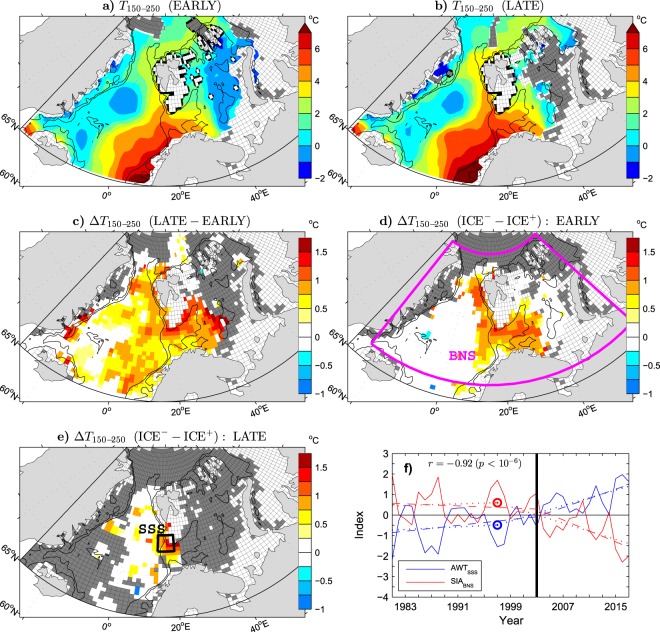
.

In Table 1, in the 5^th^ row of the 7^th^ column, under the header "*p*_LATE_",

"0.001"

should read:

"0.01"

Additionally, in Table 3, where the 3^rd^ row of the 3^rd^, 5^th^ and 7^th^ column, under the header "CSS" contains an incorrect font style,

"**0.93**", "**0.92**" and "**0.84**"

should read:

"***0.93***", "***0.92***” and "***0.84***"

As a result of the error in Figure 1b, in the "Relation to Hemispheric Variability in the Sea Ice Cover" section,

"A difference between the leading modes of the Arctic and Barents Sea SIC variability is in their association with SIC anomalies in the Greenland Sea, which is more significant for the Arctic mode (compare the patterns in Figs 3a and 1b). This difference should mainly reflect a change in the contribution of the Greenland Sea SICs to the total sea ice cover in the Barents/Nordic Seas region. In fact, unlike the Barents Sea, the Greenland Sea experienced most of its sea ice decline already during the EARLY period^58^ (see the thick black and blue lines in Fig. 2b for comparison of the mean location of the wintertime ice edge in the EARLY and LATE periods, respectively). This feature explains a slightly better (nearly perfect) agreement between PC1_SIC−A40_ and PC1_SIC−BS_ in the LATE period (*r* = 0.99), when SIC anomalies are small in the Greenland Sea but large in the Barents Sea, than in the EARLY period (*r* = 0.93). It is also consistent with a slightly stronger sea ice decline in the EARLY period associated with PC1_SIC−A40_ than PC1_SIC−BS_ (Fig. 3c, dashed lines) and with the selection of the winter 1997/98 as the onset time for the sea ice decline in PC1_SIC−A40_ (blue circle in Fig. 3c) by the objective OT detection method. Nevertheless, even in the case of PC1_SIC−A40_, most of the sea ice decline is due to the abrupt shifts in the LATE period."

should read:

"Given the very high correlation between PC1_SIC-A40_ and PC1_SIC-BS_, the associated patterns of SIC anomalies mirror one the other (compare the lobes in the Barents and Greenland Seas in Figs 3a and 1b). The lobe in the Greenland Sea is significant in both patterns. However, covariability between SIC anomalies in the Barents and Greenland Seas is moderate, as suggested by a relatively small sea ice extent in the Greenland Sea in the winter 2003/04 when the sea ice extent in the Barents Sea was large (see the thick black contour in Fig. 1b). The maximum correlation of local SIC values in the Greenland Sea with the total sea ice cover in the Barents Sea region (the SIA_BS_ index in Fig. 1c) is 0.6 (*p* < 0.01). A slightly stronger sea ice decline in the EARLY period associated with PC1_SIC-A40_ than PC1_SIC-BS_ (Fig. 3c, dashed lines) probably reflects a later but more abrupt transition to sea ice loss in the Barents Sea than elsewhere. The objective OT detection method selects the winter 1997/98 instead of the winter 2003/04 as the onset time for the sea ice decline in PC1_SIC-A40_ (see the circles in Fig. 3c)."

In the Methods section,

"(78˚-82˚N, 25˚-70˚E)"

should read:

"(76˚-82˚N, 25˚-50˚E)"

Also in the Methods section,

"The climatological mean values are calculated by weighted averaging of the local averages *T*_*l*_ at stations *s*_*l*_ found inside a circular domain of influence with the radius *R*_0_ around the centre of the given grid cell. Local inverse distance weighting is employed with weights *w*_*l*_ = (*R*_0_^2^ - *d*_*l*_^2^)/(*R*_0_^2^ + *d*_*l*_^2^), where *d*_*l*_ is the distance of station *s*_*l*_ from the cell centre."

should read:

"The climatological mean values are calculated by weighted averaging of the local averages *T*_*l*_ at stations *s*_*l*_ found inside a circular domain of influence with the radius *R* around the centre of the given grid cell. Local inverse distance weighting is employed with weights *w*_*l*_ = (*R*^2^ - *d*_*l*_^2^)/(*R*^2^ + *d*_*l*_^2^), where *d*_*l*_ is the distance of station *s*_*l*_ from the cell centre. The distance of one degree of latitude (about 111 km) is selected as *R*."

Additionally,

"The serial correlation in the time series is taken into account by employing an effective sample size defined as *N*_*eff*_ = *N*_0_(1 - *r*_*a*_*r*_*b*_)/(1 + *r*_*a*_*r*_*b*_), where *N*_0_ is the length of the series while *r*_*a*_ and *r*_*b*_ are the lag-one autocorrelations of the correlated series *a* and *b*^83^. The statistical significance of linear trends is estimated with a one-tailed Student's *t*-test."

should read:

"The serial correlation in the time series is taken into account by employing an effective sample size (*N*_*eff*_). For the correlations between the indices of climate variability listed in Table 1, *N*_*eff*_ is computed using Eq. (30) from ref. ^83^ and a biased estimator of the autocorrelations. For the correlations between the observed and predicted time series in Table 3 and to estimate the confidence level of the anomalies in the regression maps, a simplified formula for *N*_*eff*_ based on Eq. (31) from ref. ^83^ is employed, namely, *N*_*eff*_ = *N*_0_(1 - *r*_*a*_*r*_*b*_)/(1 + *r*_*a*_*r*_*b*_), where *r*_*a*_ and *r*_*b*_ are the lag-one autocorrelations of the correlated series *a* and *b* of length *N*_0_. The statistical significance of trends in the EARLY and LATE periods is estimated using a one-tailed Student's *t*-test. The *t*-statistic is based on data obtained by fitting a two-segment continuous piecewise linear model to the time series over the full ESO period with the breakpoint in the winter 2003/04 (summer 2003 in the case of the AWT_SSS_ index)."

And finally,

"PEV is positive if the regression model is better than the climate reference model (*F* = 0 in our case). It becomes unity for a perfect model, that is, when σ^2^*(F* - *T*) = 0, and minus unity for a random forecast.''

should read:

"PEV is positive if the forecast model is better than the reference model (mean value of *T*) and becomes unity for a perfect forecast (*F* = *T*). PEV can take a negative value when the employed model is utterly inadequate."

